# #*funnypoliticians*: How Do Political Figures Use Humor on *Twitter*?

**DOI:** 10.3389/fsoc.2022.788742

**Published:** 2022-04-01

**Authors:** Andrés Mendiburo-Seguel, Stéphanie Alenda, Thomas E. Ford, Andrew R. Olah, Patricio D. Navia, Catalina Argüello-Gutiérrez

**Affiliations:** ^1^Department of Psychology, Andres Bello University, Santiago, Chile; ^2^Department of Sociology, Andres Bello University, Santiago, Chile; ^3^Department of Psychology, Western Carolina University, Cullowhee, NC, United States; ^4^The Junkin Group, LLC, Philadelphia, PA, United States; ^5^Center for Latin American and Caribbean Studies, New York University, New York, NY, United States; ^6^Department of Political Science, Universidad Diego Portales, Santiago, Chile; ^7^Department of Psychology, Universidad Internacional De La Rioja, Logroño, Spain

**Keywords:** evaluation of politicians, favorability toward politicians, politicians' humor, political evaluation, social networks

## Abstract

Social media has increased its popularity among politicians. If they wish to succeed in the political arena, politicians need to present themselves to citizens as attractive individuals through these platforms. This study examined how politicians present themselves using humor on *Twitter*. We analyzed *tweets* (*n* = 6,443) from 27 politicians to determine their use of different types of humor and its relationship with age, gender, or political position. We also present changes in humor use in relation to the publication of a political survey in which politicians who were part of this study were evaluated. Results showed politicians' use of humor is relatively low in frequency and primarily aggressive. Politicians who are male, younger, and in the opposition tend to use more aggressive humor. We discuss the results considering the role of aggressive humor in political messages. Based on the analyses of *tweets* and the publication of the survey, we propose as a hypothesis for future studies that politicians' use of humor on *Twitter* could be affected by the publication of these kinds of surveys.

## Introduction

Politicians have long been aware of the power of humor to endear themselves with their constituents, to communicate their messages in a persuasive and memorable way, and to mock their opponents (e.g., Yarwood, 2001; Stewart, 2012). Indeed, U.S. Presidents Kennedy and Clinton each appointed joke writers to their speech writing teams, as they believed that well-crafted humor would make them seem closer to their audiences (Gardner, 1994; Rhea, 2012).

With the advancement of internet-based social media outlets and smartphone technology, politicians have increasingly used platforms such as *Twitter* to post political messages (Baum, 2005; Moy et al., 2005; McGregor, 2018). Accordingly, researchers have begun to systematically study the use of social media for communicating political messages (e.g., Gerodimos and Justinussen, 2015; Borah, 2016; Jungherr, 2016; López-Meri et al., 2017; Bullock and Hubner, 2020).

The present study contributes to this line of research by examining how politicians present themselves using humor on the social media platform, *Twitter*. The use of humor in public addresses by politicians is not a new phenomenon; however, there is little research on politicians' humor as a way of communication via social media, with some notable exceptions that have considered it either generically (e.g., Bullock and Hubner, 2020), or tangentially (e.g., Gerodimos and Justinussen, 2015; Borah, 2016; Jungherr, 2016; López-Meri et al., 2017). Thus, we conducted an exploratory descriptive study using 6,443 *tweets* from 27 Chilean politicians to determine how often these politicians use different types of humor (i.e., aggressive, self-deprecating, affiliative). We also investigate demographic differences in humor usage, as well as explore how humor usage varies before and after the publication of a national public opinion survey.

## Humor and Politics

Humor is viewed as a socially desirable and positive characteristic (Martin and Ford, 2018). Research suggests that humor benefits the persuasiveness of a message through a number of mechanisms: by drawing attention to the message (e.g. Hansen et al., 2009), improving message recall (Cline and Kellaris, 2007), biasing thoughts in favor of the persuasive arguments of the message and perceptions of the communicator's credibility (Nabi et al., 2007; Eisend, 2011; Stewart, 2011), and mobilizing emotions effectively (Wodak, 2015). Regardless of the specific mechanisms at play, humor is shown to help its users exert social influence (Yarwood, 2001; Nabi et al., 2007; Young, 2008).

Politicians commonly use humor not only to persuade but also to (a) communicate positive personal qualities (intelligence, social position, status), (b) generate greater recall of their message, and (c) build emotional connections with their constituents. Based on Martin et al. (2003) framework, there are three broad types of humor based on the target of the joke: aggressive humor (i.e., humor targeting others), self-deprecating humor (i.e., humor targeting one's self), and affiliative humor (i.e., benign/positive humor with no real target). However, studies addressing politicians' use of humor have largely only considered the use of two particular types: aggressive humor and self-deprecating humor (Becker, 2012; Stewart, 2012).

Aggressive humor (i.e., other-directed hostile humor) uniquely communicates two conflicting messages, an explicit message of denigration against a target, and an implicit message that the denigration is not malicious because it is “just a joke” not meant to be taken seriously (Zillmann, 1983; Martin and Ford, 2018). This inherent ambiguity gives it the appearance of social acceptability, thus averting the criticisms that serious denigration would incur (Bill and Naus, 1992). Accordingly, Verhulsdonk et al. (2021), found people were less critical of humorous vs. serious attacks on politicians.

A number of theories outline the mechanisms and contexts for which aggressive humor is positively received. The Social Identity Theory, for example, posits that individuals compare their in-groups with out-groups to build their social identity. To create a more positive identity and obtain positive distinctiveness, people highlight the positive characteristics of their in-groups and the negative attributes of the out-groups (Tajfel and Turner, 1986). From this framework, research demonstrates that experiencing aggressive humor targeting an outgroup enhances social identity, while humor targeting one's ingroup diminishes social identity (e.g., Abrams et al., 2015; Ford et al., 2020). Additionally, the Disposition Theory (Zillmann and Cantor, 1976) proposes that the response to a humorous stimulus depends on the recipient's affective disposition or attitude toward the targeted person or group (McGhee and Lloyd, 1981). That is to say, if a person has an unfavorable attitude toward a particular target, then that person will find humor disparaging that target funnier. As an example from politics, Becker (2014) found that people who disliked a prominent Republican figure in the U.S. were more likely to appreciate humor directed against the Republican Party; similarly, people appreciated humor against the Democratic Party more to the extent they disliked Barack Obama (a Democrat). Finally, aggressive humor affects the acceptance of the expression of prejudice. As proposed by the Prejudiced Norm Theory (Ford and Ferguson, 2004), people high in prejudice against a group perceive humor targeting that group as indicative of a social norm of tolerance of discrimination toward the targets of that humor. That is, it expands the norm of what is acceptable or appropriate and facilitates the manifestation of negative prejudice. Research demonstrates that such humor can increase tolerance of discrimination against the humor's target, as well as encourage personal discrimination and even violence against the target group (for a review, see Ford and Olah, 2021). Regardless of which specific theory holds most true, they all posit that aggressive humor can have real power depending on how it is used.

Research on non-humorous communications shows that when politicians express anger, they are perceived as competent, higher in status, but more unlikeable (Tiedens, 2001). Consistently, the use of aggressive *humor* has been proposed to make people seem more intelligent, more powerful, and with higher status (Stocking and Zillmann, 1976; Fine and deSoucey, 2005), and even elicit positive emotions (Ortigueira-Sánchez and Cárdenas-Egúsquiza, 2021).

On the downside, aggressive humor can also make people seem unpleasant or disruptive (Cann et al., 2016) and less sincere (Derks and Berkowitz, 1989), probably because of the relationship between this style and low agreeableness (Mendiburo-Seguel et al., 2015) and low social skills (Yip and Martin, 2006). For example, Bitterly et al. (2017) observed in a series of experiments that in cases where humor was unsuccessful (inappropriate jokes), perceived status and competence are lower. Similarly, Baumgartner et al. (2015) found that compared to a no-humor control condition, watching a video of a politician engaging in other-disparaging humor led to lower evaluations and a lower likelihood of voting for him. As opposed to this, affiliative humor, which seeks to positively enhance relationships with others without denigration of others (Martin et al., 2003), has been found to have effects on perceptions of higher intelligence and sincerity (Derks and Berkowitz, 1989).

Self-deprecating humor involves humorous attempts that target oneself, like self-mockery. Increasingly, politicians have started to use it more (Becker and Waisanen, 2017). Although some studies have shown that it has no effects on public opinions about them (Becker, 2012), others have found links with higher favorability and higher probability of voting for certain political figures (Baumgartner, 2007; Baumgartner et al., 2015). Such humor could positively help politicians, because by presenting their own defects in a socially acceptable manner, they can generate greater identification (Meyer, 2000), a common goal of politicians when using humor in other contexts such as presidential debates (Rhea, 2012). At the same time, they can demonstrate that they have not only the ability to see their own flaws and defects, but also the value and social status to be able to accept them publicly (Greengross and Miller, 2008; Stewart, 2011).

Although aggressive and self-deprecating humor have been the two main focus of study, there is a third humor type that could be used by politicians. As an instance of humor, affiliative humor is directed toward others in a positive way (Martin et al., 2003). Critical to understanding comic interactions on *Twitter*, there is an intention behind affiliative humor to have fun with others in a friendly way. That is, affiliative humor functions as a beneficial type of interaction and a facilitator of positive relations that enhances social relationships (Martin and Ford, 2018).

## Humor and Politicians on Social Networks

*Twitter* has become a useful tool for politicians to show interest and personally connect to people, thereby influencing their audience's general evaluation of their credibility and image (Jackson and Lilleker, 2011; Lassen and Brown, 2011; Jungherr, 2016). Politicians may also use social media to determine which communications have a more positive effect and help their visibility (D'heer, 2018); personalized messages can improve message recognition and recall and bolster the politicians' image among people higher in affiliative tendencies (Lee and Shin, 2012; Lee and Oh, 2013). That is, *Twitter* may allow politicians to foster feelings of intimacy and social presence through the development of unidirectional and para-social relationships that shorten psychological distance between citizens and politicians (Lee and Shin, 2012; McGregor, 2018).

Politicians use humor in their messages on social media (Papacharissi and Oliveira, 2012), although evidence varies widely on the extent they use it and its outcomes. For instance, in his campaign for the U.S. elections of 2008, 11.5% of all messages by Barack Obama on Facebook had humorous emotional content (compared to a 3.1% of John McCain), and in 2012 that number rose to 21.2% (Mitt Romney did not use humor). Obama's humorous posts garnered a higher number of interactions via “likes” or “shares” on his Facebook page (Borah, 2016). In other cases, such as the 2009 Federal Elections in Germany, the number of humorous messages varied between much lower numbers: 0.1% and 0.6% (Jungherr, 2014). Similarly, during the Spanish electoral campaign of 2016, humor was the least used content by political parties and politicians on *Twitter*, although Pablo Iglesias used it a 5.9% of the time, and one of his *tweets* (a joke about his relation with communism) was the most re*tweet*ed of the election (López-Meri et al., 2017). In summary, although not all politicians embrace humor to the same extent in their social media communications, it seems that using humor in online interactions has political impacts.

### The Role of Politicians' Political Affiliation, Gender and Age on Their Use of Humor on *Twitter*

The adoption of *Twitter* accounts seems to be related with strategic concerns instead of political affiliation or ideology (Vergeer and Hermans, 2013; Quinlan et al., 2018), although there is evidence that suggests that more extreme (liberal or conservative) political figures use *Twitter* more than those that are less-extreme (Straus et al., 2013). In the case of humor use, research tends to focus on how people with different ideologies appreciate it more than how they produce or use it. In that line, more liberal individuals tend to enjoy more non-sense humor (Ruch and Hehl, 1988), while conservatives enjoy humor to a lesser extent in general (Bonanno and Jost, 2006). When understanding humor as a trait, or ways in which individuals show humor in their everyday life (or *humor styles*), research shows that people with left-wing orientation are more likely to use affiliative and aggressive humor, but they are not overall more humorous (Kfrerer et al., 2021); further, liberals tend to be higher in the cynic comic style, or the depreciation of commonly acknowledged values through humor (Mendiburo-Seguel and Heintz, 2020).

Regarding gender, it is not clear if male or female politicians have a higher use of *Twitter* or other social media (though research with general populations suggest gender is not associated with having a *Twitter* account; Straus et al., 2013). Some studies have found that women candidates are more active in its use (Evans et al., 2014), while other have observed the opposite (Hemphill et al., 2013).

Although research on gender and social media use is mixed, there is much evidence indicating gender differences and similarities between different humor-related characteristics. For example, men tend to use and enjoy aggressive humor more than women; there is no clear pattern regarding overall humor production, though some research suggests that humor communication is related with gender roles (for a review of gender differences in humor-related traits, see Hofmann et al., 2020). In fact, the stereotype of an “ideal sense of humor” refers to men in several different countries (Tosun et al., 2018).

Concerning age, evidence shows that younger politicians use new media (and specifically *Twitter*) to a larger extent (Straus et al., 2013; Vergeer and Hermans, 2013), probably because they have more familiarity with new technologies (Gibson and McAllister, 2006). Another explanation is press coverage: since older politicians tend to receive greater traditional press coverage (Straus et al., 2013), maybe the reason for younger politicians to use *Twitter* is to become more recognizable and not necessarily to be better evaluated.

Humor production tends to decrease with age (for a review, see Greengross, 2013), as do aggressive comic styles such as irony, sarcasm, and cynicism (Mendiburo-Seguel and Heintz, 2020); affiliative styles show a less consistent pattern across studies (Martin et al., 2003; Ruch et al., 2018; Mendiburo-Seguel and Heintz, 2020; Dionigi et al., 2021).

## The Present Study

This study contributes to humor and political communication research in two main ways. First, we seek to understand different political contexts, specifically by studying politicians in Chile. The study of variables related to politics has been largely based on specific Western countries; even then, there are differences even between similar political contexts and political systems (Boxell et al., 2020). For this, the case of Chile offers an interesting example, considering the political changes that finally led to the social outburst of October 2019, the emergence of a more organized extreme right or the revitalization of the Communist Party and left-wing figures and movements, such as the coalition known as *Broad Front* [Frente Amplio] (Lindh et al., 2019). In such a context, it is possible to think that differences between these political actors and/or the need to differentiate themselves could make them use humor as a communication tool.

Our study also adds up to the existing literature by considering other aspects of politicians' use of humor on *Twitter* that have not been considered before. Previous research has focused on how frequently politicians *tweet*, the percentage of their *tweets* that intend to be humorous, or has considered humor as a generic variable not distinguishing between different types. Thus, the present study contributes to the existing research by examining *how* politicians use different types of humor on *Twitter*, also considering a third humor strategy: affiliative humor.

In particular, we explore the following research questions:

RQ1: To what extent are different types of humor used by politicians on *Twitter*?

RQ2: Are there any differences in the use of humor between politicians of different gender, political affiliation, and age?

RQ3: Does the way in which politicians use humor vary across time, considering the publication of political surveys?

To these ends, we first describe the use of each type of humor, and compare them depending on politicians' gender, political affiliation, and age. After this, we explore in a descriptive manner how the use of humor on *Twitter* by politicians varies considering two periods in time (before and after the publication of results of the 83rd Public Opinion National Study; that is, before and after politicians receive critically updated information about how they are perceived by citizens).

## Materials and Methods

In this study, we considered *tweets* from 27 Chilean political figures, which were selected based on the results of the 83rd Public Opinion National Study (Centro de estudios públicos [Centre of Public Studies]., 2019), a survey that assessed opinions regarding each figure.

Although the dataset initially included 28 politicians, one was not part of our analysis because her *Twitter* account did not have any activity for at least 2 years. In all the cases, the complete list of *tweets* was retrieved via the *Twitter* API and using the *rtweet* package (Kearney, 2019) for R (R Core Team, 2020).

We retrieved all the *tweets* (total *n* = 47,063) from each politician's account since their accounts were first activated, including replies and re*tweets* with comments by the politician, but excluding re*tweets* without comments. However, considering the differences on these dates, we decided to work with *tweets* from 2016, since that was the year when at least half (59%) had an account (total *n* = 37,773). We therefore selected a stratified random sample considering political affiliation, gender, and age as strata (*n* = 6,443, margin of error = 1.1%, 95% CI).

Once the *tweets* were obtained, two coders were asked to read and classify each of them according to the variables of interest (presence or absence of humor, and in the case of humorous *tweets*, if they implied the aggressive, affiliative, self-deprecating, or self-enhancing humor style). Both coders were students from a research methods course and were previously trained and given examples separately. The training consisted of giving and explaining the definitions of each type of humor. As a general rule, we asked the coders to classify as humorous any *tweet* that was intended to entertain or comically attract attention, emphasizing that such intent had to be directly attributable to the person who wrote the *tweet*. Given that the study's objective was related to humor creation and not the comic phenomenon in a general way, only *tweets* where the politician had been the creator of humor were considered. Others, such as responses like “*hahaha*,” “*how funny!*,” or “*what a laugh!*” were left out unless the context indicated that they were intended ironically.

We also trained coders to determine what type of humor each *tweet* was. To illustrate them, the first author gathered examples of *tweets* from the database that met the characteristics of each type. As aggressive humor, we asked them to search for any humorous *tweet* that sought to ridicule or attack a person, idea, or group, whether through irony, sarcasm, satire, or cynicism [according to the objectives of each style described by Ruch et al. (2018)]. In the case of self-deprecating humor, the target had to be the same politician alluding to his or her defects, errors, or faults comically. In the case of self-enhancing humor, *tweets* had to be a clear allusion to their own positive characteristics. Finally, in the case of affiliative humor, *tweets* had to have an affiliative and well-intentioned intention, according to the definition of Martin et al. (2003).

As *tweets* could include different contexts, such as replies, judges were also given the link to each of them. Coders were given 1 month to classify the *tweets*, after which their classifications were given to a third rater, who was asked to decide in the cases where there was no agreement. The third rater could also decide that classification was difficult enough so as to leave a particular *tweet* as “no agreement.”

After this procedure, Kappa coefficients were computed to assess inter-rater reliability, obtaining satisfactory results in all cases: 0.93 for total use of humor (95% CI 0.92–0.95), 0.94 for affiliative humor (95% CI 0.91–0.98), 0.72 for aggressive humor (95% CI 0.69–0.75), 0.79 for self-defeating humor (95% CI 0.66–0.92). No cases of self-enhancing humor were found on the analyzed sample. Table 1 shows an example of each type of humorous tweets.

**Table 1:** Examples of humorous tweets.

**Affiliative humor**
*We inaugurated the 1st “Popular Optic” of the Valparaíso region! It will be open to everyone no matter what their health system is. The differences are at “plain sight.”*
**Aggressive humor**
[As a reply to a user that insulted him] *Don't tweet when you're drunk. People tell me it's your usual state, take care of yourself*
**Self-deprecating humor**
[As a reply to a user that tweeted “If there's one thing I like about him, it's that he is not a thermocephalus”] *Only that?* 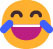

### Considered Variables

***Total use of humor*** was computed as the percentage of the total *tweets* by each politician that was intended to be funny: [*N humorous tweets/(humorous* + *serious tweets)]* × 100

***Humor types*** were computed as the percentage of the total *tweets* by a politician that used each of them: [*N tweets containing each humor style*/*(humorous* + *serious tweets)]* × 100

Politicians were classified depending on political affiliation (left wing or right wing), gender (female or male), and age (see Table 2).

**Table 2:** Description of considered political figures.

**Name**	**@**	**Affiliation**	**Gender**	**Age**
Andrés Allamand	*allamand*	R	M	63
Álvaro Elizalde	*alvaroelizalde*	L	M	50
Andrés Chadwick	*andreschadwickp*	R	M	63
Jacqueline Van Rysselberghe	*coca_vanr*	R	F	54
Daniel Jadue	*danieljadue*	L	M	52
Mario Desbordes	*desbordes*	R	M	51
Evelyn Matthei	*evelynmatthei*	R	F	66
Fuad Chahín	*fchahin*	L	M	42
Felipe Kast	*felipekast*	R	M	42
Felipe Larraín	*felipelarrain*	R	M	61
Gabriel Boric	*gabrielboric*	L	M	33
Giorgio Jackson	*GiorgioJackson*	L	M	32
Guillermo Teillier	*gteillier*	L	M	76
Alejandro Guillier	*guillier*	L	M	66
Heraldo Muñoz	*HeraldoMunoz*	L	M	71
Hernán Larraín Matte	*hernanlarrain*	R	M	44
José Miguel Insulza	*insulza*	L	M	76
Jorge Sharp	*JorgeSharp*	L	M	34
José Antonio Kast	*joseantoniokast*	R	M	53
Beatriz Sánchez	*labeasanchez*	L	F	48
Ricardo Lagos Weber	*lagosweber*	L	M	57
Joaquín Lavín	*LavinJoaquin*	R	M	66
Michelle Bachelet	*mbachelet*	L	F	68
Marcela Cubillos	*mcubillossigall*	R	F	52
Manuel José Ossandón	*mjossandon*	R	M	57
Sebastián Piñera	*sebastianpinera*	R	M	69
Jaime Quintana	*senadorquintana*	L	M	52

*R, Right; L, Left; M, Male; F, Female; T, Total number of tweets; S, Sample*.

## Results

### Uses of Humor by Politicians and Differences Between Genders, Political Affiliation, and Age

Of the considered politicians, 5 (18.5%) were women and 22 (81.5%) were men, with a mean age of 55.48 years (*S.D*. = 12.51). The number of female politicians was lower than the number of male politicians, which is in line with the proportion of women in positions of power in politics (26.0%; United Nations Development Programme, 2020).

Table 3 shows total *tweets*, humorous *tweets*, and used humor types for each group. The analysis of the *tweets* shows that 7.0% of them had humorous intent: 5.2% were aggressive, 1.2% were affiliative and 0.2% were self-deprecating. The proportion of aggressive *tweets* was significantly higher than the proportion of affiliative and self-deprecating *tweets* (both *p's* < 0.001), and the proportion of affiliative *tweets* was significantly higher than the proportion of self-deprecating *tweets* (*p* < 0.001).

**Table 3:** Percentage of tweets that contain humor depending on gender, and political affiliation.

	**Gender**		**Political affiliation**		**Total**
	**Female**	**Male**	**Right**	**Left**	
*N*	5	22	13	14	27
Total tweets containing humor	3.8%	7.8%	7.3%	6.7%	7.0%
Affiliative humor	1.2%	1.2%	1.6%	0.9%	1.2%
Aggressive humor	1.7%	6.2%	5.5%	5.0%	5.2%
Self-deprecating humor	0.2%	0.0%	0.2%	0.2%	0.2%

As shown in Table 3, male politicians used more humor than female politicians in general [$\chi {{}^{\text{2}}}_{\left(1\right)}$ = 27.659, *p* < 0.001, ϕ = 0.07]. When breaking down by humor type, we see these gender differences are specific to the use of aggressive humor [$\chi {{}^{\text{2}}}_{\left(1\right)}$ = 44,268, *p* < 0.001, ϕ = 0.08]. There were no gender differences in the use of affiliative humor or self-deprecating humor.

In the case of age, there was a negative correlation with the total use of humor [*r*__*s*_(25)_ = −0.50, *p* < 0.01], the use of aggressive humor [*r*__*s*_(25)_ = −0.42, *p* = 0.03], and the use of self-deprecating humor [*r*__*s*_(25)_ = −0.43, *p* = 0.03]. That is, older politicians used less humor overall, and specifically used less aggressive and self-deprecating humor. The correlation between age and the use of affiliative humor was non-significant.

Regarding political affiliation, there were no differences between left-wing and right-wing politicians regarding total use of humor [$\chi {{}^{\text{2}}}_{\left(1\right)}$ = 0.575, *p* = 0.448], aggressive humor [$\chi {{}^{\text{2}}}_{\left(1\right)}$ = 0.244, *p* = 0.621], and self-deprecating humor [$\chi {{}^{\text{2}}}_{\left(1\right)}$ = 0.013, *p* = 0.908]. Right-wing politicians tended to use more affiliative humor than left-wing politicians [$\chi {{}^{\text{2}}}_{\left(1\right)}$ = 5.653, *p* = 0.017, ϕ = 0.03].

We also analyzed how the use of humor varied depending on what coalition was in government. During 2016 and 2017 the left-wing was the majority coalition, and during 2018–2019 it was the right coalition. During the time they were the opposition, right-wing politicians *tweet*ed more humorous *tweets* (8.7%) than left-wing politicians [4.2%, $\chi {{}^{\text{2}}}_{\left(1\right)}$ = 14.290, *p* < 0.001, ϕ = 0.09], specifically aggressive *tweets* (7.9 and 1.9%, respectively; $\chi {{}^{\text{2}}}_{\left(1\right)}$ = 35.237, *p* < 0.001, ϕ = 0.15]. When the left-wing politicians were the opposition, there were no differences in the total number of humorous *tweets*, but they *tweet*ed fewer humorous *tweets* with affiliative content (0.7%) than the right-wing politicians [1.8%, $\chi {{}^{\text{2}}}_{\left(1\right)}$ = 9.822, *p* < 0.01, ϕ = 0.05] and more humorous *tweets* with aggressive content [6.5 and 4.9%, respectively; $\chi {{}^{\text{2}}}_{\left(1\right)}$ = 5.960, *p* = 0.015, ϕ = 0.04]. Collectively, the opposition party appears to use more aggressive humor than the majority party, regardless of which specific political affiliation is in charge.

### Use of Humor Over Time and Possible Relations With Opinion Surveys

To give an in-depth examination of the use of humor over time, we decided to do an exercise based on favorability. As a point of comparison, we considered the publication of the 83rd Public Opinion National Study, given that all the politicians that were considered for our study were evaluated in it. However, we do not propose a causal relationship between the survey and how politicians use humor; instead, we present this as an exploratory exercise.

**Figure 1:**
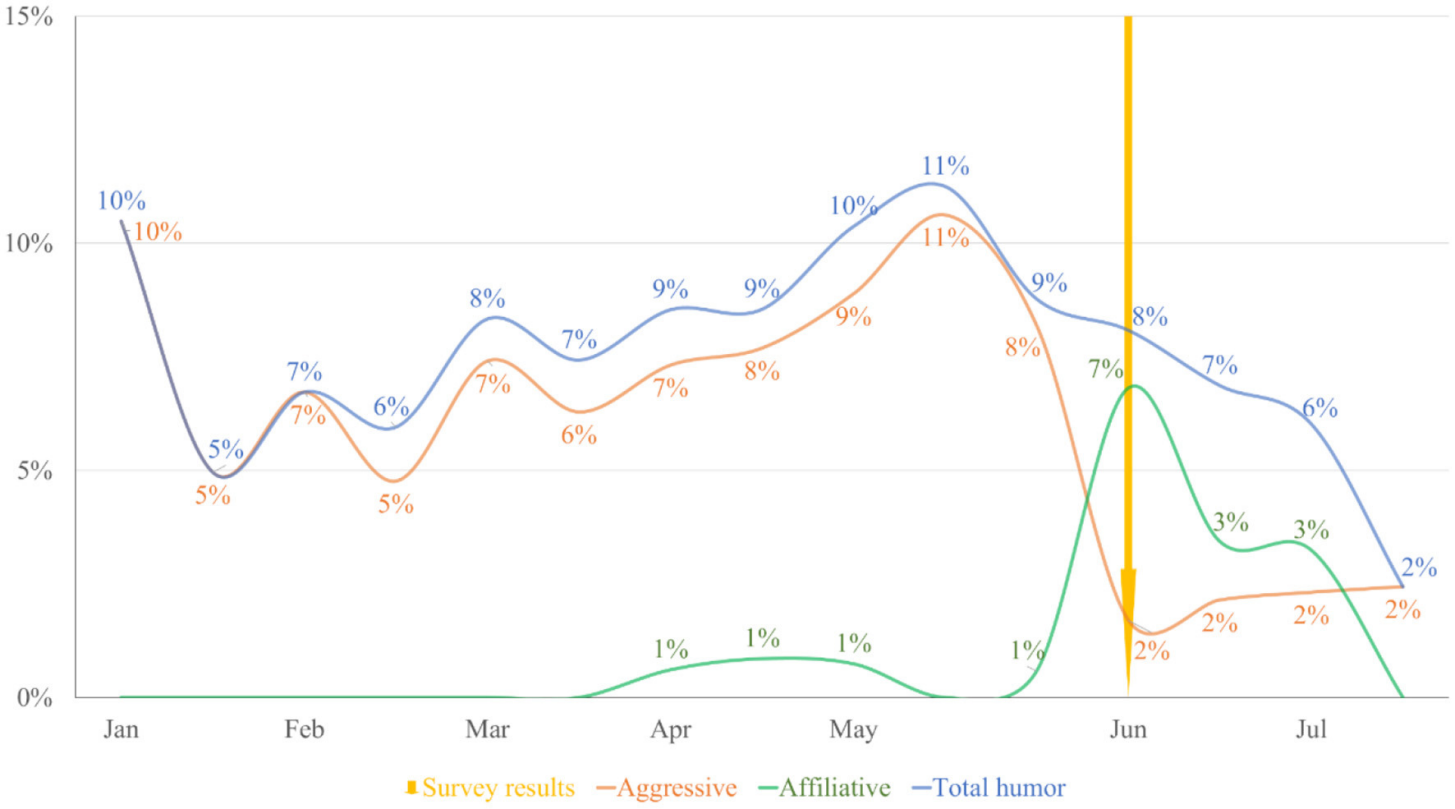
Percentage of humorous, affiliative, and aggressive tweets of all the considered political figures. We present rounded percentages with no decimals to facilitate the interpretation of the figure.

Figure 1 presents the use of humor from January 1st to July 19 of 2019 to have an overview, but comparisons are made considering seven weeks before (April 22–June 12) and after (June 13–July 20) the publication of the 83rd Public Opinion National Study (June 13). We used these periods because the obtaining of the *tweets* was on July 21 and we wanted to compare considering the same number of weeks before and after the publication. Given the low number of self-deprecating *tweets*, we did not consider them in these analyses. Table 4 shows the number of humorous, affiliative, aggressive, and self-deprecating *tweets* before and after the 83rd Public Opinion National Study was published.

**Table 4:** Total tweets and tweets containing humor 3 weeks before and 3 weeks after survey results.

	**Before**	**After**	**Total**
Total tweets	466 (100.0%)	490 (100.0%)	956 (100.0%)
Humorous tweets	47 (10.1%)	30 (6.1%)	77 (8.1%)
Affiliative humor tweets	2 (0.4%)	15 (3.1%)	17 (1.8%)
Aggressive humor tweets	43 (9.2%)	11 (2.2%)	54 (5.6%)

*Percentages in parenthesis are in relation to total tweets*.

Considering all the politicians, although the number of *tweets* was similar, the percentage of total humorous *tweets* and aggressive *tweets* dropped significantly after the publication of the 83rd Public Opinion National Study (*z* = 2.20, *p* = 0.028 and *z* = 4.63, *p* < 0.001, respectively). In contrast, affiliative *tweets* increased significantly (*z* = 3.10, *p* < 0.01). Figure 1 displays these trends in 2-week intervals over time.

## Discussion and Conclusions

Although the use of social media as a vehicle for political communication has been a subject of interest previously, there has not been a specific focus on the use of humor. Considering both have been documented as effective political tools, the results of this study shed novel light on politician's use of humor on social media.

At a descriptive level, it is necessary to recall that all of the politicians that were considered for this study had a *Twitter* account and that -except for one- it was active, indicating that it has become a popular and almost mandatory way of communication for them. Regarding the main focus of this study, we found that politicians use humor in a relatively small number of their *tweets*, with the most common being aggressive. In fact, the use of the other types was much less frequent, which is in line with other studies (e.g., Stewart, 2011) that have observed that self-deprecating humor is not a predominant style among political candidates, although it is well-received by people.

It is possible that the higher use of aggressive humor is related to the attraction of attention. As Bode et al. (2016) propose, in the political arena *tweets* should attract attention and two ways of accomplishing that are through humor and insults/controversial statements. In fact, previous studies have found that in the case of Facebook, comments with negative emotional content generate more reactions and are shared more than other comments (Bene, 2017). Considering that, among politicians, traditional press coverage is higher for those with more seniority (Straus et al., 2013), it is possible that lesser-known politicians use humor (and especially aggressive humor) in order to attract attention and, in that way, become more recognizable. This idea is supported by the present study's finding that politicians' use of aggressive humor decreases with age, though future research might explicitly explore whether attention-seeking motives mediate this relationship.

When considering gender, we observed that male politicians use humor more than females, and especially aggressive humor. This is a common pattern in several studies unrelated to politics (Martin and Ford, 2018), so it is likely that this finding reflects general gender differences, such as men's preference for sexual or aggressive humor (Hofmann et al., 2020). Indeed, it has been observed that women prefer less offensive patterns of political behavior online (Maximova and Lukyanova, 2020), and the systematic review by Hofmann et al. (2020) concluded that there could be social pressures that discourage women's use of aggressive humor.

Regarding political affiliation, previous studies in the U.S. context have concluded that there are no significant differences between Republicans and Democrats regarding how they use humor focused on the in-group or out-group (Stewart, 2011). In our case, it is interesting to note that the use of different types of humor is in fact more related with the *role* (as part of the Government or the opposition) of political coalitions more than party affiliation. At a general level, right-wing politicians use more affiliative humor, although the effect size is very small, but when considering their role, aggressive humor appears as the key element: when politicians are part of the opposition, they use more aggressive humor. This could be expected since aggressive humor is a useful way to express dissent and attack others, so it could be expected that those who are part of the opposition attack the government through humor, probably aiming to be read by those of the same political position. Using humor as a vehicle of aggression adds a playful frame and ambiguity to situations, so people may interpret the user's intentions in different ways (Martineau, 1972; Meyer, 2015). Thus, by creating an ambiguous and “harmless” message, humor allows an attack that provides a “just joking” defense to politicians if this attack is not well-received (Kuipers, 2006). However, it should not be understood “just as a joke,” as explained by the Prejudiced Norm Theory (Ford and Ferguson, 2004). Attacking through jokes not only serves the opposition to express discontent with the government but could also mobilize people from more extreme political positions and, in this way, promote the manifestation of rejection toward the government's ideas and contrary positions in general. As a result, it could contribute to polarizing citizens.

In the case of how the use of humor varies over time, our results should be considered exploratory and in no way a sign of causation. Overall, we observed that the proportion of humorous *tweets*, both aggressive and affiliative, was relatively stable before the publication of the survey: aggressive *tweets*' proportion varied between 5 and 11%, and affiliative *tweets*' proportion was low (between 0 and 1%). Although aggressive humor showed variations before the publication of the survey, there was a notorious drop in its use after that point in time. The opposite happened in the case of affiliative humor, which had a peak at 7% to then drop to 3% 2 weeks later. Does this mean that the publication of the survey was the cause of these changes? Based on our data, it is impossible to say this, so these results should be interpreted as a trend more than causation. However, based on the analysis of these trends, a question arises: might politicians adapt their communication strategies (specifically on *Twitter*) in response to citizens' evaluations of them as indicated by polling data? Our results could serve as a starting point to study this in the future, considering comprehensive models like the two-step flow model Lazarsfeld et al. (1960). This model posits that politicians communicate indirectly to citizens through “Opinion Leaders,” or people that inform themselves about politics, are present in various social structures, and transmit political information to other people; in this way, these opinion leaders serve as a mediator between politician's communications and citizens' perceptions, and thus might influence how a politician's humor is received. This model could help analyze how politicians use *Twitter* to reach primary and secondary audiences with their *tweets* and re*tweets* (Karlsen, 2015; Vaccari and Valeriani, 2015).

## Limitations and Future Steps

This study addresses a line of research that has become increasingly relevant to understand how political figures engage the public, explicitly observing humorous political communication in a cultural context that is not usually considered in academic political analyses. In order to have a better understanding of the implications of these results, there are some limitations to consider.

First, the nature of this study does not make it possible to establish causality. This is not only because of the characteristics of observational data but also because the period we used to observe variations in humor was long enough to suspect that several other variables could have affected it.

On *Twitter*-based studies, machine learning techniques allow researchers to analyze more data without having to individually code *tweets*. We think that it would be useful to apply this in cases like the ones we present in this study (acknowledging, however, that humor recognition -and specifically aggressive types like sarcasm or irony- using such technology still presents difficulties that human-based content analysis can help alleviate; Sykora et al., 2020). That way, mixed techniques emerge as a good option to continue this line of research and get information from a higher number of politicians that could add heterogeneity to the sample. Finally, we think that other interesting analysis should be conducted. Specifically, we consider that focusing in particular on both the radical right and left (as opposed to more moderate politicians) would be interesting. These future inquiries could explore the use of particular types and targets of aggressive humor associated with these extreme sensibilities and evaluate its political efficacy. This could shed new light on the facets of personalization of politics through social media, on how a particular “rhetoric style” influences the way politicians are perceived by their supporters and opponents, and humor's specific role in this relationship.

## Data Availability Statement

The dataset will be available upon request after the publication of a second article based on it. Requests should be directed to andres.mendiburo@unab.cl.

## Author Contributions

AM-S conceived the original idea, worked out the technical details, analyzed the data, and wrote the first version of the manuscript. SA, TF, AO, and PN helped developing the theoretical formalism and development of the research questions, contributed to the interpretation of the results, the discussion of the results, and to the final manuscript. CA-G contributed to the interpretation of the results, the discussion of the results, and provided corrections to the final manuscript. All authors contributed to the article and approved the submitted version.

## Funding

This study was funded by the Fondo Nacional de Desarrollo Científico y Tecnológico—Fondecyt Grant No. 1210556 and Universidad Andrés Bello, Fondo Jorge Millas Grant No. DI-03-19/JM.

## Conflict of Interest

AO was employed by The Junkin Group, LLC. The remaining authors declare that the research was conducted in the absence of any commercial or financial relationships that could be construed as a potential conflict of interest.

## Publisher's Note

All claims expressed in this article are solely those of the authors and do not necessarily represent those of their affiliated organizations, or those of the publisher, the editors and the reviewers. Any product that may be evaluated in this article, or claim that may be made by its manufacturer, is not guaranteed or endorsed by the publisher.
